# Supplementary material for: Label-Free Imaging of Catalytic H2O2 Decomposition on Single Colloidal Pt Nanoparticles Using Nanofluidic Scattering Microscopy
Source: ACS Nano. 2023 Oct 17;17(21):21030–43. doi: 10.1021/acsnano.3c03977 (PMC10655234; doi:10.1021/acsnano.3c03977)
Supplement: Supplementary file 1 — nn3c03977_si_001.pdf [file nn3c03977_si_001.pdf]

Supplementary Material

For

Label-free Imaging of Catalytic H_2O_2 Decomposition on Single Colloidal Pt Nanoparticles using Nanofluidic Scattering Microscopy

*Björn Altenburger[1], Carl Andersson[1], Sune Levin[2], Fredrik Westerlund[2], Joachim Fritzsche[1] and Christoph Langhammer[1]**

[1]Department of Physics, Chalmers University of Technology; SE-412 96 Gothenburg, Sweden

[2]Department of Biology and Biological Engineering, Chalmers University of Technology; SE-412 96 Gothenburg, Sweden

*Corresponding author: clangham@chalmers.se

Section I: Supplementary figures

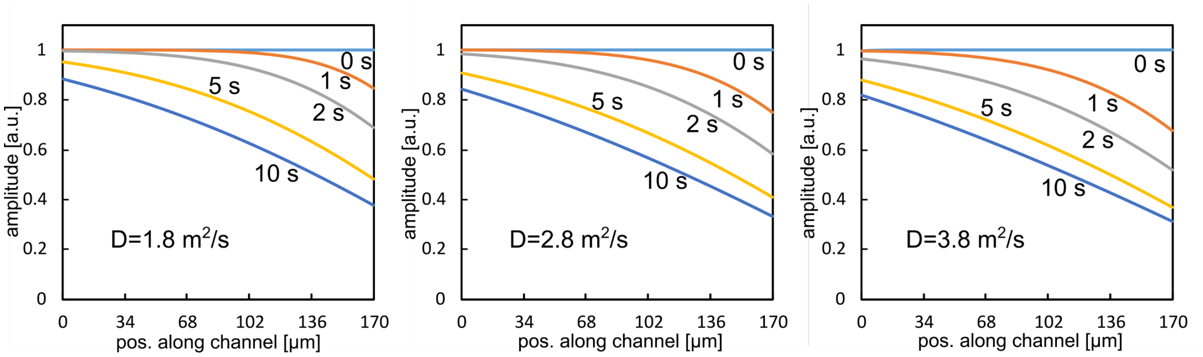

Figure S1. Calculated time evolution of H_2O_2 concentration profiles in a quadratic nanochannel with 150 nm side length for bulk diffusion constants D = 1.8 m^2/s, D = 2.8 m^2/s and D = 3.8 m^2/s.

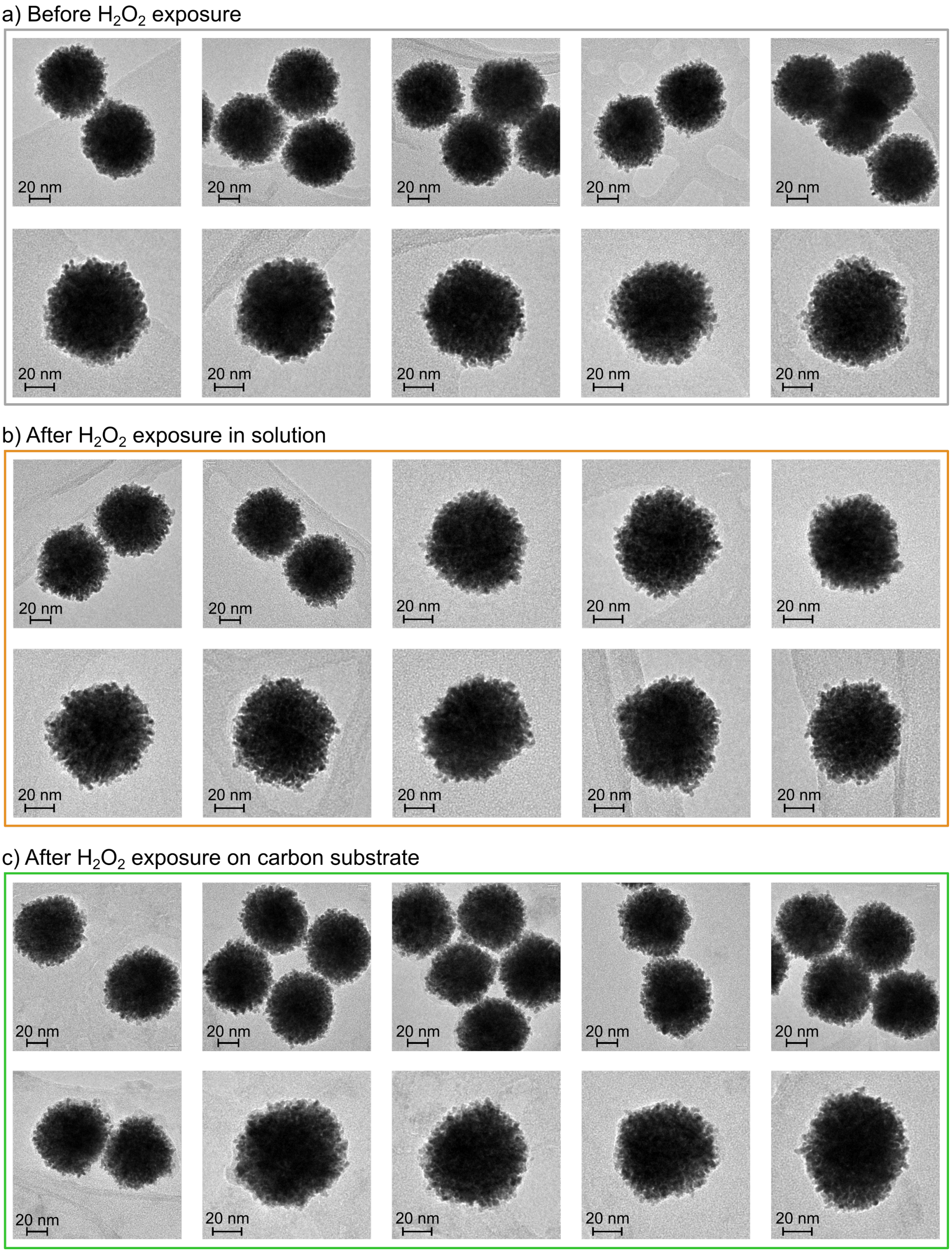

Figure S2. Selection of TEM-pictures of the colloidal Pt particles used in our experiments before and after exposure to reactions. All images were obtained by drop casting the particles onto holey carbon TEM grids. a) Pt particles as extracted from the storage solution before they have been in contact with 30% H_2O_2 in water. b) Platinum particles after exposure to H_2O_2 decomposition reaction conditions in aqueous solution for ca. 5 minutes. c) Platinum particles that have been subjected to H_2O_2 after drop casting them onto holey carbon. While the particles seem quite similar, it is evident that they are not identical and vary in surface structure and size. There is also no effect by H_2O_2 on the particles visible, only the carbon support seems rougher in the TEM sample that was treated with H_2O_2.

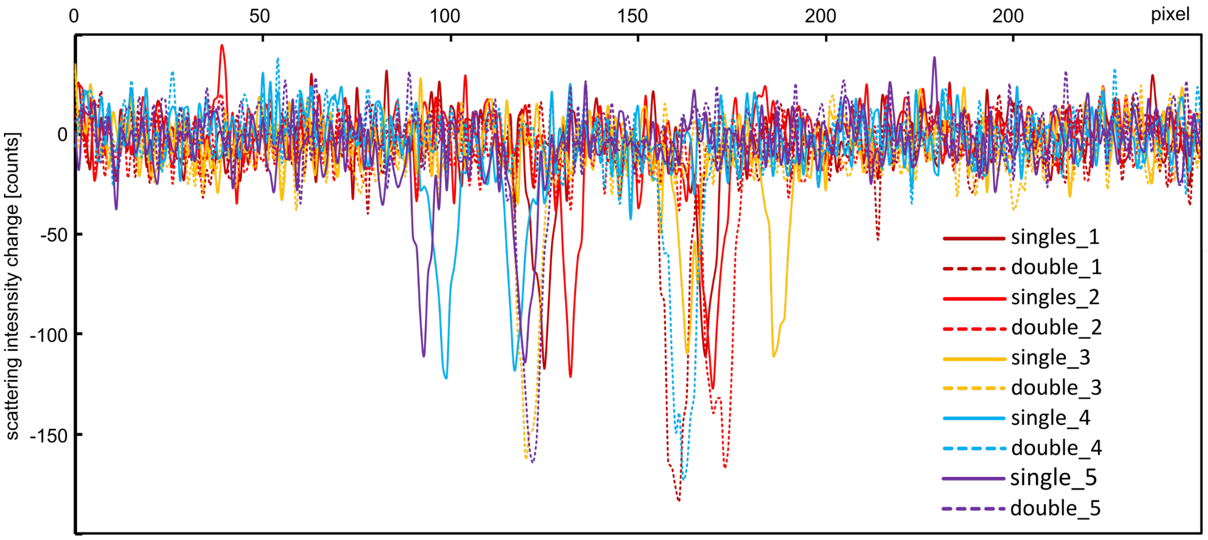

Figure S3. Experimental light scattering intensity changes measured along the channel direction (expressed as pixel-# along the x-axis) for nanochannels containing two diffusing Pt particles that transiently combine in a diffraction limited spot and from a "dimer". The color-coded curves show the scattering intensity difference between channel and particles in their separated and "dimer" state for each channel. They reveal a decrease in intensity of about 40 % for the dimers compared to their corresponding single particles.

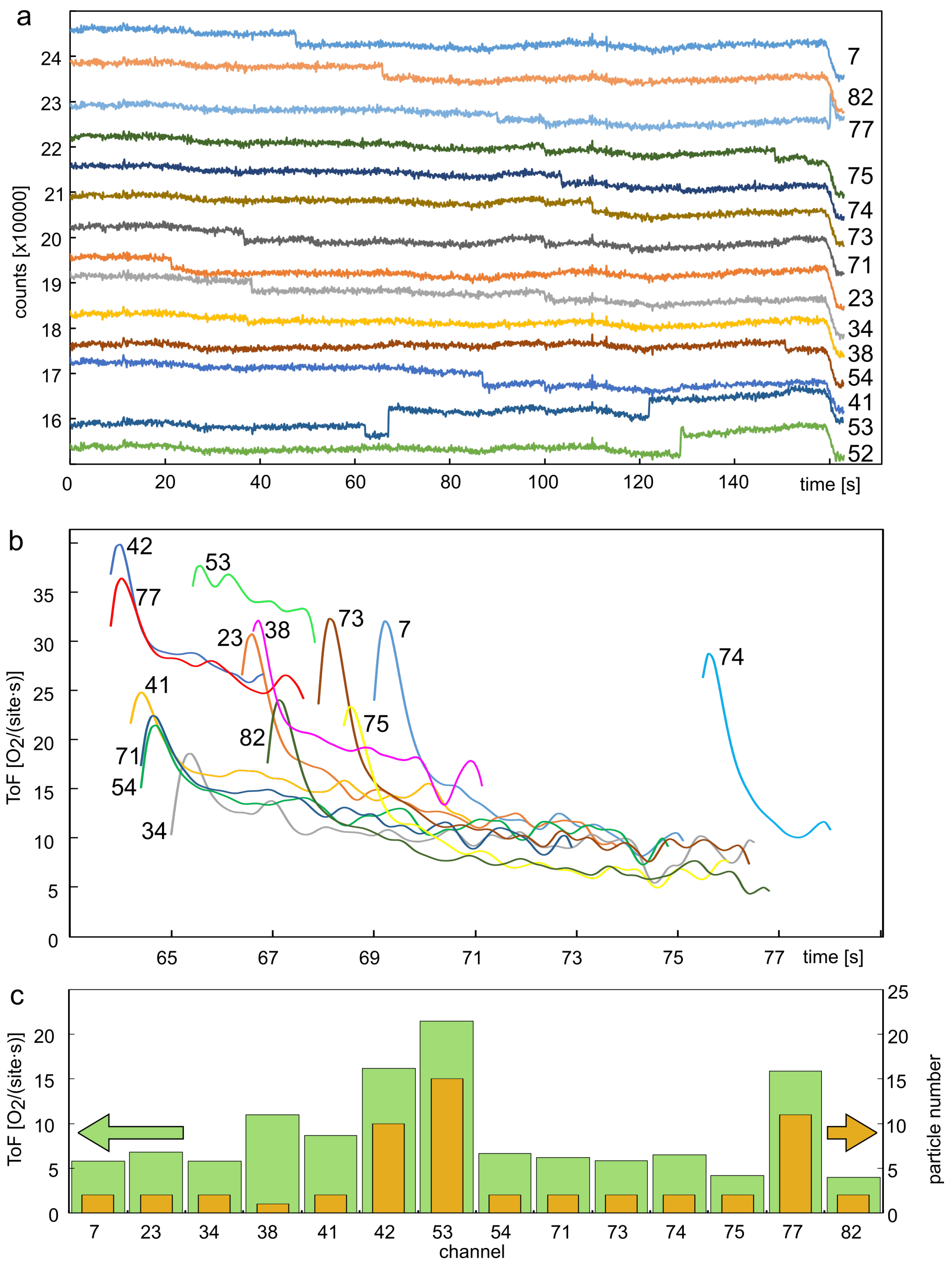

Figure S4. Pt particle counting and bubble evaluation. a) Integrated scattering intensity time traces recorded for the trap area for the measurement shown in **Figure 4** in the main text for all channels that show bubble formation according to Scenario I. The arrival of individual particles can be seen as distinct steps to lower intensity. However, it can also be seen that when multiple particles accumulate, the scattering intensity at the trap instead increases and becomes higher than in the empty reference channels numbered 42, 53 and 77. B) ToF traces for the channels shown in a) for an H_2O_2 concentration of 30% in water. c) Comparison of the counted (and for the largest numbers estimated) number of particles in each channel and the corresponding ToF derived from the BES.

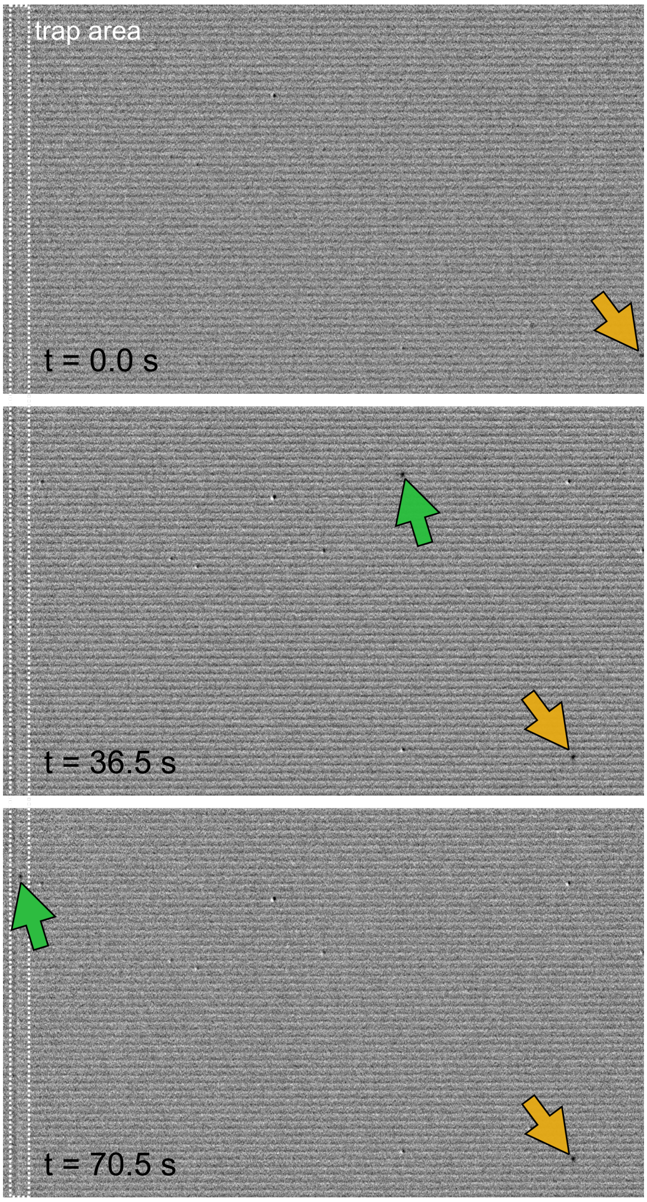

Figure S5. Citrate coated Pt particles getting stuck in nanochannels before the trap. The particle marked with orange arrow enters the field of view at t = 0s as it is pushed through the channel but gets stuck due to electrostatic interaction with the wall at t = 36.5 s and remains in the same position at t = 70. 5 s. The particle marked with a green arrow gets transiently stuck at 36.5 s before eventually reaching the trap at 70.5 s.

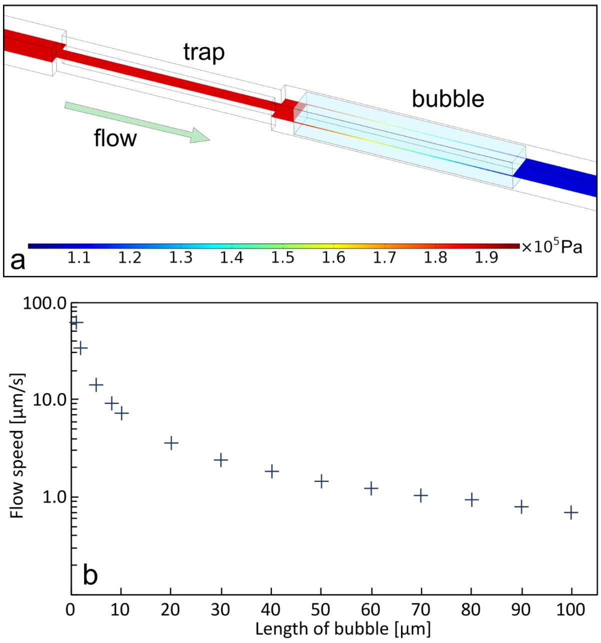

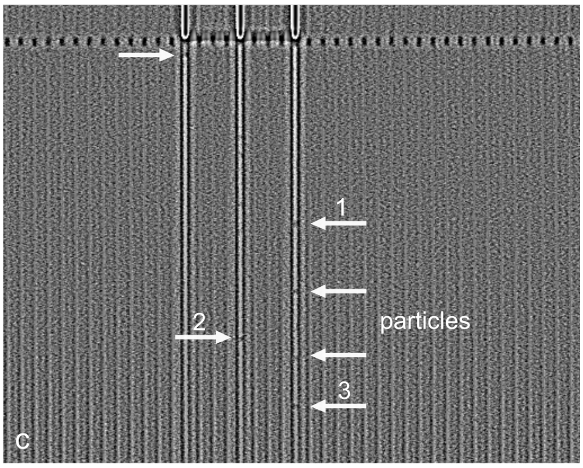

particle	Δt [s]	Δs [µm]	v [µm/s]
1	5	38.24	7.65
2	4	21.00	5.25
3	1.3	10.34	7.95

Figure S6. Water flow speed assessment for the nanochannels at 2 bar inlet pressure. a) Comsol simulation of the pressure drop at the position of a 1 µm long bubble that occupies 75.5% of a 150 nm x 150 nm nanochannel and that is positioned after the constriction that traps the nanoparticles (slice through the middle shows pressure as color gradient). While the pressure remains constant throughout the trap, it is decreased by 1 bar after the bubble. b) Simulated flow speed inside the nanochannel as a function of the length of a bubble that is growing inside that channel. The maximum length of the bubble is 170 µm, which corresponds to the length of the nanochannel after the constriction. c) Measurement of the flow speed inside nanochannels of identical dimensions as in the simulation obtained by tracking of the movement of Pt nanoparticles (arrows) induced by the flow. The differential dark-field image shows how particles can be seen moving out of the channels when the pressure at the inlets of the chip is inverted (flow from top to bottom). The three channels that stand out are filled with a bubble on the other side of the trap (top). Tracking several particles while the same pressure is applied as during bubble formation allows an estimation of the flow speed when a bubble is filling the channels. The table lists the correspondingly obtained results from three different Pt particles.

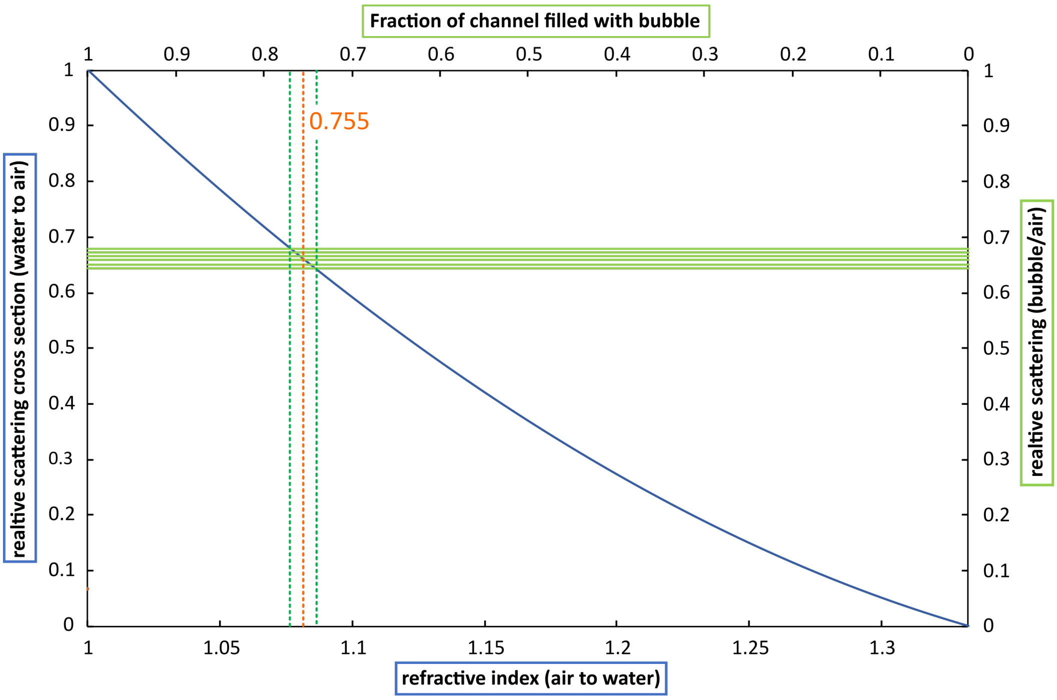

Figure S7. Comparison to estimate the fraction of the nanochannel that is filled with gas when a bubble is filling the channel. Blue x- and y-axis: Calculated relative scattering cross section between water and air (as a mimic of O_2 in the gas phase) plotted as a function of refractive index systematically changing from air to water. The relative scattering cross section of a completely air-filled channel has been set to 1 and for a completely water filled channel it is set to 0. Green x- and y-axis: Measured scattering from a set of O_2 bubble-filled channels scaled relative to the scattering of the same channels filled with air using the same scaling as for the simulated data. The comparison of the experimental with the theoretical curve yields the estimate that the gas bubble occupies the channel volume to ca. 75.5%.

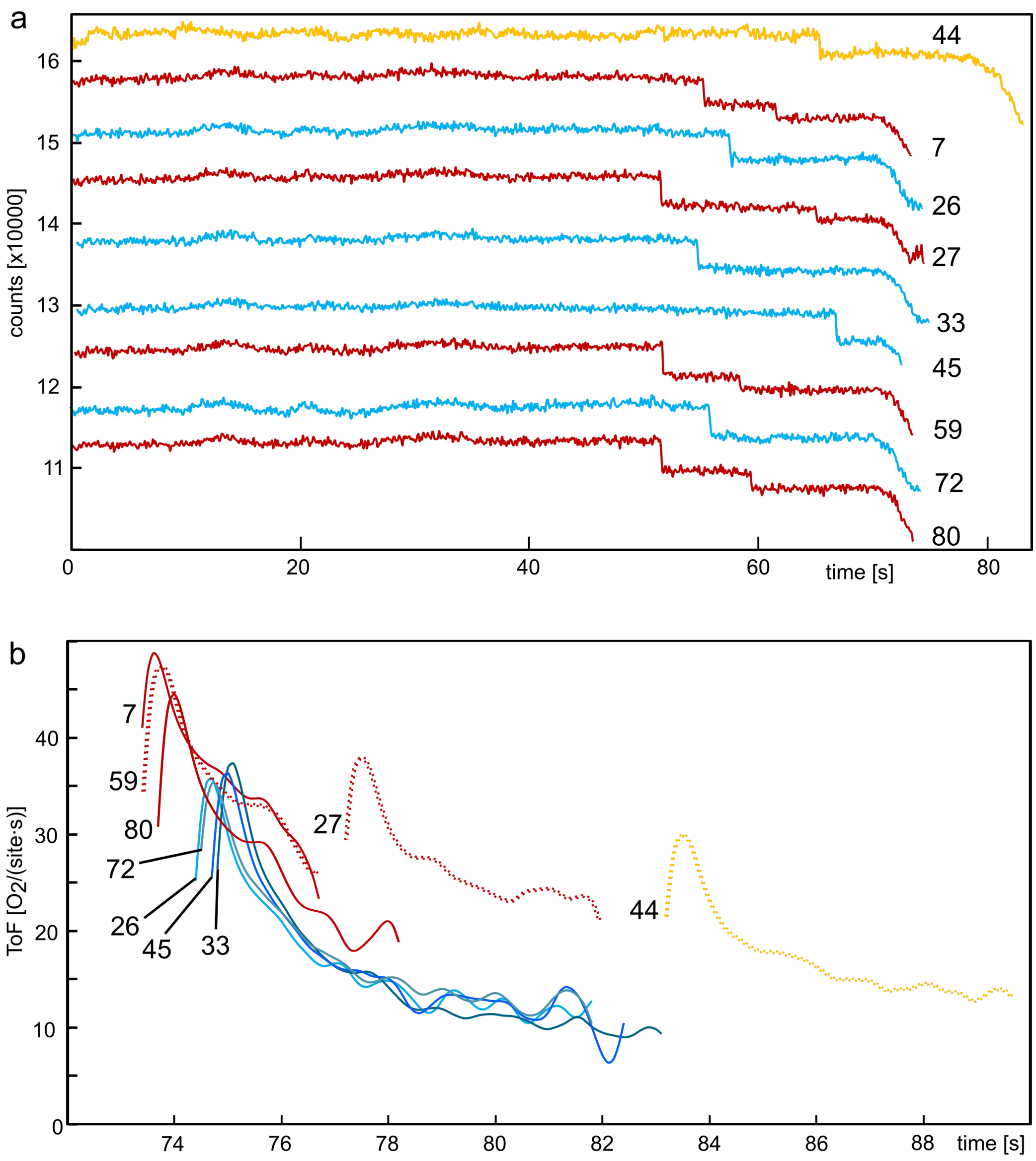

Figure S8. a) Integrated scattering intensity time traces of the trap area for the measurement shown in **Figure 5** in the main text for all channels that show bubble formation according to Scenario II. The arrival of single or multiple particles can be seen as steps in the intensity time trace. Red curves represent channels with two particles getting trapped (seen as two individual steps), blue and yellow curves represent channels where a single particle is trapped. b) ToF traces calculated from the BES for Scenario II bubbles forming in the same channels as analyzed for trapped particles in panel a). Solid ToF traces mark cases where the particle(s) remained stationary at the trap, whereas dashed ToF traces mark cases where particles were pushed out by the developing bubble. The color code is the same as in a). Blue and red ToF traces were measured for a 15% H_2O_2 reactant solution and the yellow trace was measured for a 30% H_2O_2 reactant solution.

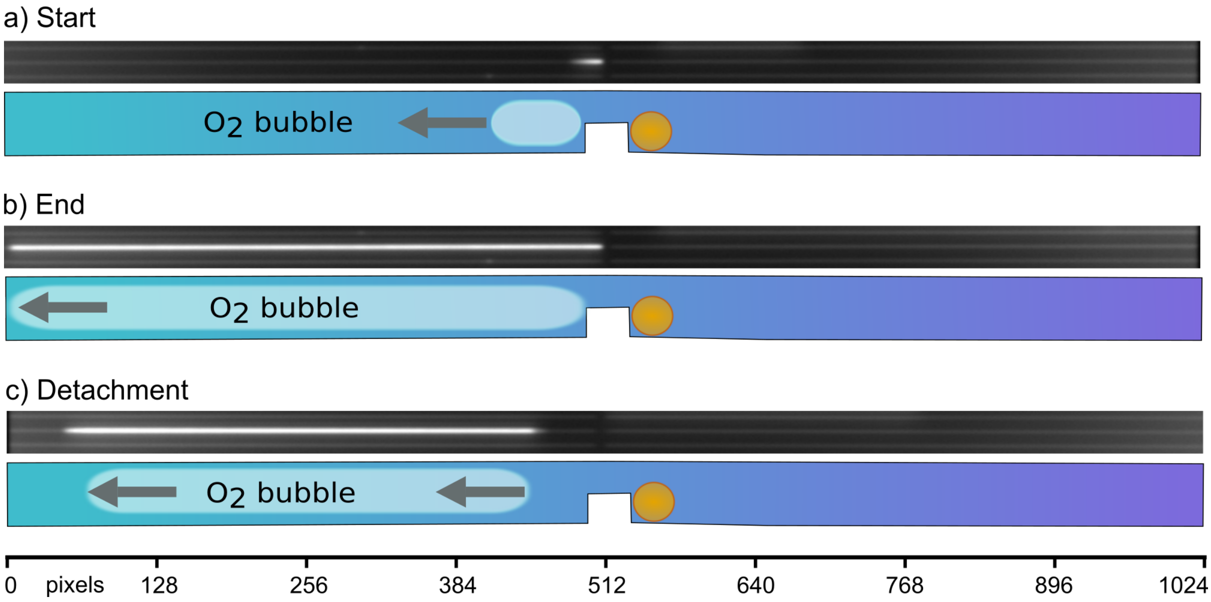

Figure S9. Start and end points of a BES trace. A) The BES trace starts as soon as a visible bubble starts to form, that is, when the brightness of some pixels along the nanochannel is reaching a certain threshold. The BES trace then corresponds to the number of pixels over this threshold over time. b) The tracing of the bubble extension speed is ended when the bubble reaches the edge of the field of view of the camera, since from this point forward the number of bright pixels remains constant. c) In some cases, the bubble detaches from the trap and moves through the nanochannel. This will also be regarded as an end to the BES trace since the bubble is not growing anymore and since the connection to the particle is lost.

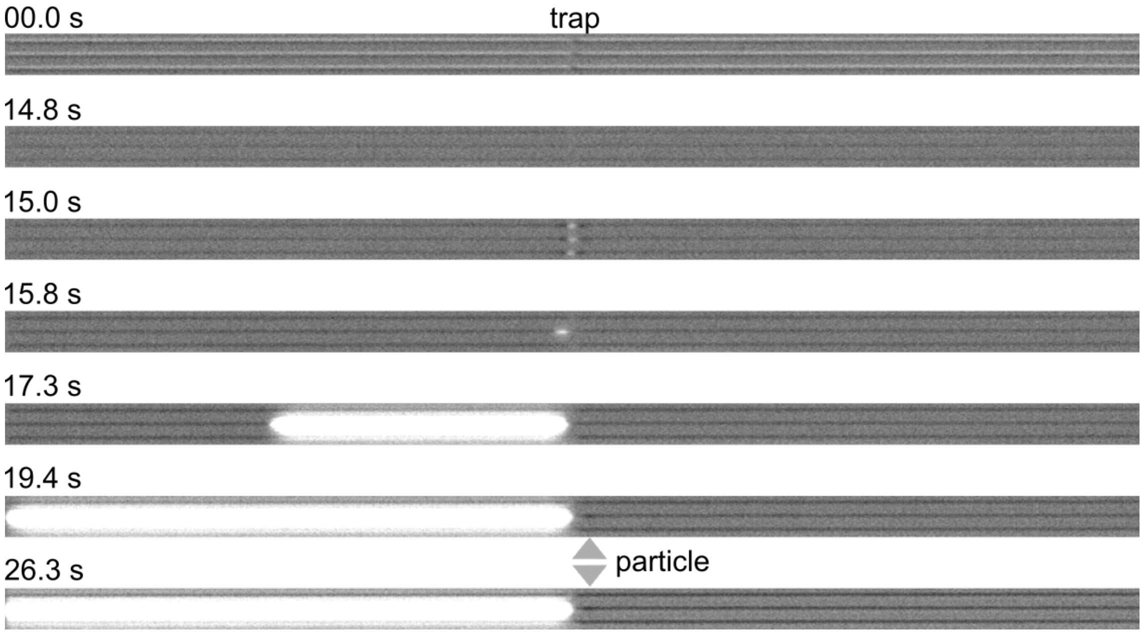

Figure S10. **Still images of characteristic time points in movie SV3.** For this movie, a frame obtained at 14.5 s (immediately before H_2O_2 entered the channel) has been subtracted from all subsequent frames to highlight subsequent changes in scattering intensity. 0.00 s) Water-filled channels with the trap area in the middle. The particle is already in place in the nanochannel in the center. 14.8 s) The 30% H_2O_2 solution is starting to fill the channel when the flow of water was switched off. This is visible by the darker channels and the overall slightly darker right side of the image. 15.0 s) Disturbances cause movement of the whole chip, which makes the trap area shortly visible in these differential images. Reasons for these disturbances can be the pressure switch from the water inlet side to the H_2O_2 inlet side of the chip or the touching of the pressure switches which transfers to the chip via the pressure pipes. 15.8 s) The O_2 bubble starts to form on the side of the trap that is opposite the particle in the center channel after the H_2O_2 solution has been flushed in. 17.3 s) The bubble has extended about half the way to the edge of the field of view. 19.4 s) The bubble has reached the edge of the field of view, such that the BES trace is terminated. Since the convective flow is now largely stopped by the bubble blocking the channel, the particle drifts a short distance away from the trap (arrows). 26.3 s) The bubble has extended beyond the field of view. On the particle side, the center channel and its neighboring channels have the same low brightness, indicating so that they are filled with about the same concentration of H_2O_2.

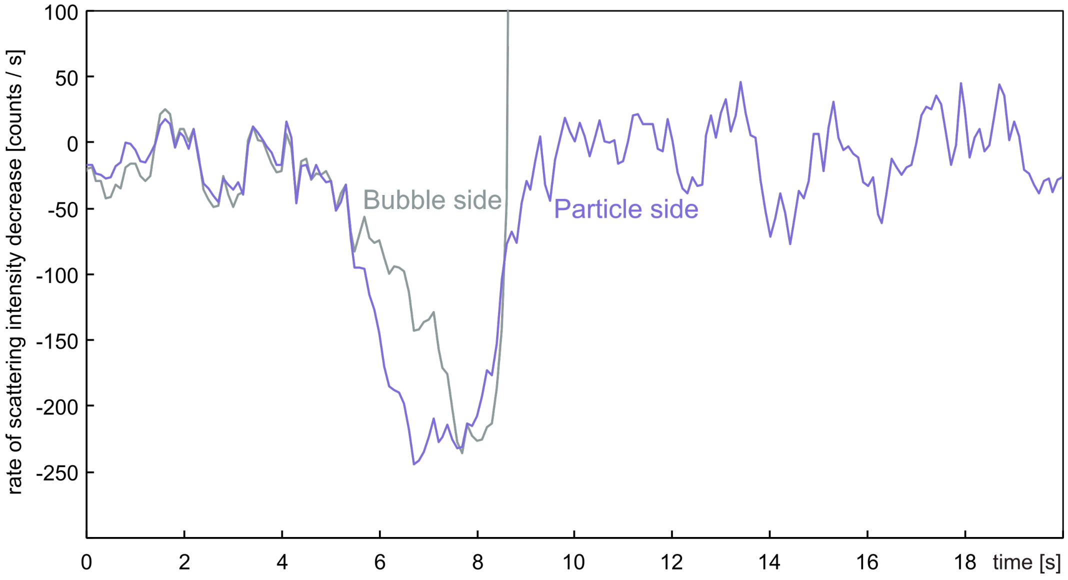

Figure S11. Time derivative of the channel scattering intensity traces shown in Figure 5a in the main text that reveals a clear shift of the peak of the derivative by about 1 s between the two separately imaged channel sections down and upstream of the particle, respectively. This indicates a time delay of about 1 second for the H_2O_2 front reaching these two channel sections.

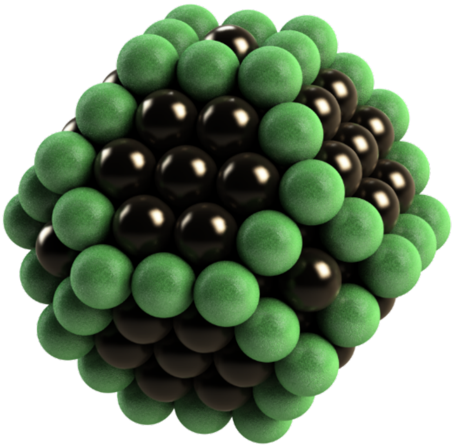

Figure S12. Atomistic representation of a Pt nanoparticle with a total of 201 atoms. In this configuration, there are 124 atoms located on the surface, whereof 60 are low-coordinated edge or corner sites, while the remaining 64 atoms are located in terraces. The fraction of edge/corner sites at the surface is thus 48.4%. Even though the size of this particle is with only 1.7 nm diameter[1] at the lower end of the observed crystallite sizes of the Pt particles used in our experiments (see **Figure 3a** in the main text), it rationalizes the edge & corner/terrace site ratio of ca. 50 % we use in our discussion as reasonable and representative for the Pt nanoparticles at hand

Section II: Supplementary Derivations

1. Theoretical calculation of nanochannel scattering cross sections

The derivation of the scattering cross section formula for a nanochannel, **Error! Reference source not found.**in the main text, is described in detail in chapter 8 in the book by Bohren and Huffman[2]. For convenience, we summarize the key steps below.

To mathematically describe the rectangular channels from the experiment, we use a cylinder of infinite length, whose diameter corresponds to the cross section of the experimental nanochannel. Accordingly, the derivation uses cylindrical coordinates as given in **Figure S10**.

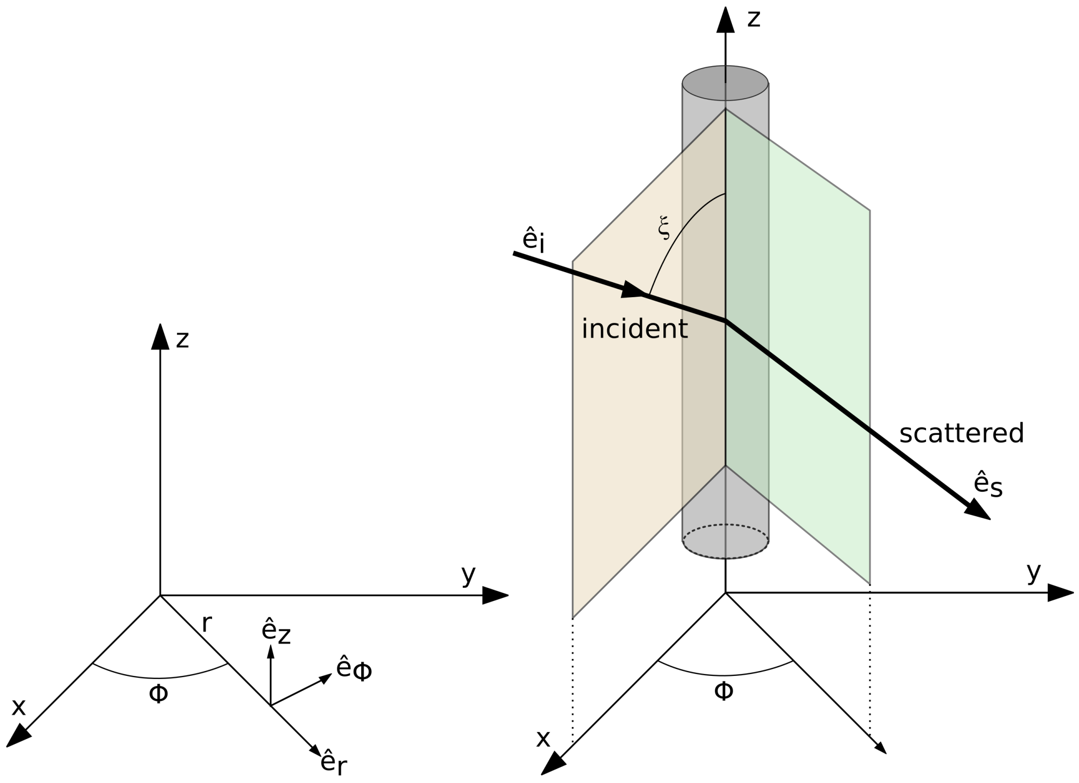

Figure S13. Cylindric coordinate system and a section of the infinite cylinder with the Poynting vector of the incident and scattered light.

The function that solves the wave equation in cylindrical coordinates can be written in a form that separates into a radial, angular and vertical part, where $h = -k\cos\zeta$ is the separation constant and $\rho = r\sqrt{k^2 - h^2}$ with k as wavenumber.

$$\psi_n(r,\phi,z) = Z_n(\rho)e^{in\phi}e^{ihz} \quad (n = 0, \pm 1, \dots)$$

Linearly independent solutions to Z_n are the Bessel functions of the first and second kind, J_n and Y_n respectively, where n denotes their integral order. The harmonic functions generated from the equation above,

$$M_n = \nabla \times (\hat{e}_z \psi_n), \qquad N_n = \frac{\nabla \times M_n}{k}$$

will be used to express the incident electric field of a plane wave $E_i = E_0 e^{ik\hat{e}_i x}$ that is incident onto a cylinder of radius a in the direction of $\hat{e}_i = -\sin\zeta\hat{e}_x - \cos\zeta\hat{e}_z$ with ζ being the angle between cylinder axis and the incident wave. For an incident electric wave that is parallel to the xz-plane, the expansion in cylinder harmonics is

$$E_i = \sum_{n=-\infty}^{\infty} [A_n M_n^I + B_n N_n^I].$$

The generating function for the cylinder harmonics in then $J_n(kr\sin\zeta)e^{in\phi}e^{-ikz\cos\zeta}$, as we need to exclude the Bessel functions of the second kind to avoid an infinite electric field at $r = 0$. Following Bohren and Huffman, the coefficients are given as

$$A_n = 0, \qquad B_n = \frac{E_0(-i)^n}{k\ \sin\zeta}$$

such that for the incident wave, when $E_n = E_0\,(-i)^n/k\sin\zeta$,

$$E_i = \sum_{n=-\infty}^{\infty} E_n N_n^I, \qquad H_i = \frac{-ik}{\omega\mu}\sum_{n=-\infty}^{\infty} E_n M_n^I$$

For the internal field, we define first the ration of refractive indexes

$$m = \frac{n_{cylinder}}{n_{surrounding}}.$$

to arrive at

$$E_I = \sum_{n=-\infty}^{\infty} E_n[g_n M_n^I + f_n N_n^I], \qquad H_I = \frac{-ik}{\omega\mu}\sum_{n=-\infty}^{\infty} E_n[g_n N_n^I + f_n M_n^I]$$

For the scattered field, we find

$$E_s = \sum_{n=-\infty}^{\infty} E_n[b_{n1}N_n^3 + ia_{n1}M_n^3], \qquad H_s = \frac{ik}{\omega\mu}\sum_{n=-\infty}^{\infty} E_n[b_{n1}M_n^3 + ifa_{n1}N_n^3]$$

Such that we now can describe the Poynting vector

$$S_s = \frac{1}{2}\,\mathrm{Re}(\boldsymbol{E}_s \times \boldsymbol{H}_s^*) \quad S_s = \frac{1}{2}\,\mathrm{Re}(\boldsymbol{E}_i \times \boldsymbol{H}_s^* + \boldsymbol{E}_s \times \boldsymbol{H})_i^*$$

and the absorption, scattering and extinction rates for the incoming light.

$$W_a = -\int_A \boldsymbol{S}\cdot\hat{\boldsymbol{n}}\, dA = W_{ext} - W_s = RL\int_0^{2\pi} (\boldsymbol{S}_{ext})_r\, d\phi - RL\int_0^{2\pi} (\boldsymbol{S}_s)_r\, d\phi$$

Comparing these rates with the projected cross section of the channel, we arrive at the scattering efficiency (for parallel incident light).

$$Q_{sca,p} = \frac{W_s}{2aLI_i} = \frac{2}{x}\left[|b_0|^2 + 2\sum_{n=1}^{\infty}(|b_n|^2 + |a_n|^2)\right]$$

The coefficients a_n and b_n can be found in the book by Bohren and Huffman[2], and we arrive at the scattering efficiencies for both parallel, and, in similar manner, orthogonally polarized incident light.

$$Q_{sca,p} = \frac{\pi^2 x^3}{8}(m^2-1)^2 = \frac{\pi^2 k^3 a^3}{8}(m^2-1)^2$$

$$Q_{sca,o} = \frac{\pi^2 x^3}{4}\left(\frac{m^2-1}{m^2+1}\right)^2 = \frac{\pi^2 k^3 a^3}{4}\left(\frac{m^2-1}{m^2+1}\right)^2$$

From those, we use $x = ka = 2\pi a/\lambda$ and as geometrical cross section $A_\emptyset = \pi a^2$ to arrive at the scattering cross sections for both polarization directions of incident light, as well as for unpolarized light, which corresponds to **Error! Reference source not found.** in the main text.

$$\sigma_{sca,p} = \frac{A_\emptyset^2 k^3 L}{4}(m^2-1)^2$$

$$\sigma_{sca,o} = \frac{A_\emptyset^2 k^3 L}{2}\left(\frac{m^2-1}{m^2+1}\right)^2$$

$$\sigma_{sca,u} = \frac{A_\emptyset^2 k^3 L}{4}(m^2-1)^2\left(\frac{1}{2} + \frac{1}{(m^2+1)^2}\right)$$

2. *Influence of oxygen solubility*

For the estimation of the influence of the O_2 solubility in water on the bubble formation in a nanochannel, we start at an average BES of 6.3 µm/s, which can be translated into an O_2 production rate of $2.5 \cdot 10^6\ O_2/s$ per particle when using **Error! Reference source not found.** in the main text.

From Figure 2d in the main text, we can estimate the flow speed of the liquid in the channel at an inlet pressure of 2 bar to be $40\ \mu m/s$. Multiplied with the channel cross section of 150 x 150 nm^2, we arrive at a volume flow of $7.84 \cdot 10^{-19}\, m^3/s$. Using now the solubility of $1.22\ mol/m^3$ and Avogadro's constant, we see that about $0.6 \cdot 10^6\ O_2/s$ could be transported away by the flow in the channel, which is about a quarter of the above estimated O_2 production rate.

This will however be only the case at the very start of the reaction, because as soon as a bubble has formed, the flow speed will be drastically decreased (see also **Figure S6**), thereby limiting the transport of dissolved oxygen and thus significantly reducing this effect.

3. Turnover frequency from bubble expansion speed

At the start, we need to derive the amount of O_2 produced per second. To do this, we assume that the bubble is rectangular as the channel and has a cross section that is the geometrical cross section of the channel times the filling factor determined in **Figure S7**.

$$A_{bub} = 0.755 A_{channel}$$

Multiplied by the BES, which has the unit of m/s, we arrive at the bubble volume expansion speed in m^3/s. Divided by the volume of 1 mol of O_2 gas, $V_{O2}^{mol} = 22.4\ l/mol$, we get the number of moles of molecular oxygen produced within 1 s.

$$R_{o2} = \frac{A_{bub}\ BES}{V_{O2}^{mol}}$$

Multiplying this with Avogadro's number, we get the absolute amount of O_2 molecules produced per second. Divided by the number of active sites N, we get the number of O_2 molecules produced per site and second, which corresponds to the turnover frequency.

$$ToF = \frac{0.755\ A_{channel}\ BES\ N_A}{V_{O2}^{mol} N}$$

References

(1) Rivera Rocabado, D. S.; Ishimoto, T.; Koyama, M. The Effect of SnO2(110) Supports on the Geometrical and Electronic Properties of Platinum Nanoparticles. *SN Appl. Sci.* **2019**, *1* (11), 1–15.

(2) Bohren, C. F.; Huffman, D. R. Rayleigh-Gans Theory. *Absorpt. Scatt. Light by Small Part.* **2007**, 158–165.
